# Educational Intervention Effects on Depression and Anxiety in Patients after Myocardial Infarction: A Randomized Controlled Trial

**DOI:** 10.3390/jcdd10070267

**Published:** 2023-06-22

**Authors:** Lazzat M. Zhamaliyeva, Damira G. Zhamankulova, Nurgul A. Abenova, Gulbakit K. Koshmaganbetova

**Affiliations:** 1 Department of Family Medicine, West Kazakhstan Marat Ospanov Medical University, Aktobe 030019, Kazakhstan; 2 Department of Internal Diseases, West Kazakhstan Marat Ospanov Medical University, Aktobe 030019, Kazakhstan; bianconeri-98@mail.ru; 3 Department of General Practice 1, West Kazakhstan Marat Ospanov Medical University, Aktobe 030019, Kazakhstan; nurgul_abenova@mail.ru; 4 Department of Master’s and Doctoral Studies, West Kazakhstan Marat Ospanov Medical University, Aktobe 030019, Kazakhstan; gulbakit66@mail.ru

**Keywords:** anxiety, cardiac rehabilitation, depression, medical education, primary care

## Abstract

Depression and anxiety in cardiovascular disease worsen the prognosis of patients. Treatments for these disorders often provide limited improvement. The present study aimed to test, for the first time, the impact of educational technology on anxiety and depressive symptoms in patients participating in a cardiac rehabilitation program. A 12-month randomized controlled trial was conducted, in which 207 patients were randomly assigned to either the experimental (n = 76) or control (n = 69) groups. The intervention involved a structured patient education program provided by medical students who had undergone specially designed training in cardiac rehabilitation. The primary outcomes were death, hospitalization, heart failure, and recurrent myocardial infarction. The study also assessed anxiety and depression. A year later, the experimental group showed a statistically significant decrease in anxiety and depression on the HADS scale, with reductions of 2.0 and 1.9 points, respectively (*p* < 0.05). The control group showed reductions of 1.5 and 1.2 points (*p* < 0.05). The difference in the Hamilton Rating Scale for Depression between the groups at 12 months was −1.29 in favor of the main group (95% CI, −0.7 to −1.88), and the standardized mean difference was 0.36 (95% CI, 0.03 to 0.69). No treatment-related adverse events were observed. The results suggest that educational interventions can have a positive impact on mental health. The study’s strengths include a structured intervention, randomization, and long-term follow-up. The limitations include the lack of blinding of study participants and a relatively small sample size.

## 1. Introduction

Cardiovascular disease (CVD) is the most prevalent non-communicable disease [1], and it is the leading cause of morbidity and mortality worldwide. Psychological consequences, such as anxiety and depression, affect up to one-third of people with CVD and are associated with an increased risk of coronary diseases, lower quality of life (QoL), and increased healthcare costs [2,3]. Therefore, the prevention and treatment of anxiety and depression are essential components of patient care [4]. Although several studies have evaluated the effect of antidepressants and psychotherapy on depression in CVD, their effect on depressive symptoms was negligible or small [5].

Cardiac rehabilitation (CR) has been shown to be effective in reducing depression [6]. Patients who completed the CR program reported higher levels of physical and mental QoL and lower levels of depression [7]. One accessible, well-received, and side-effect-free method for addressing the psychological and physical problems of patients is a well-organized educational program [8]. Given the growing number of patients and the shortage of nurses and doctors in many countries, trained medical students are recruited to work with patients, and this strategy has proven successful [9].

Providing patients and caregivers with good education and information about recovery is an essential aspect of chronic patient care [10,11]. A recent Cochrane review provides evidence that education improves the knowledge of cardiac patients and their relatives, increases satisfaction, and decreases depression rates [12]. Many patients want to receive additional knowledge about the causes and prognosis of the disease, preventive measures, the availability of public services, and informal support groups.

There is a difference between informing patients and educating them. Information refers to healthcare issues, while patient education refers to interventions that facilitate care, increase adherence to treatment, lifestyle modifications, and informed decision-making [13]. The effectiveness of teaching depends on how it is delivered [14]. This applies to recipients and service providers, so choosing educational interventions is crucial.

Educational interventions are a wide range of technologies, methods, and training tools to form competent professionals [15]. Educational interventions provide learners with the support they need to acquire skills and should address the functional, academic, cognitive, behavioral, and social skills that directly affect educational ability [16]. New educational programs, courses, and pedagogical methods aim to reform the old practices used [14]. The review of the conducted studies showed that the main limitations were the short or medium intervention period (from 8 weeks to 6 months), the often-mediated nature of the intervention (via the Internet or telephone), and the fragmentation of care (depression in patients with CVD was dealt with by separate specialists). In our project, a 12-month intervention was planned, with personal (home) visits of volunteers who guided patients for not only depression and anxiety, but also primarily for heart disease and learning new/healthy lifestyles [5].

Efforts aimed at developing patient education skills in healthcare professionals, mainly nurses, ensure long-term success in disease management [14]. In the existing system of primary healthcare in Kazakhstan, with a shortage of personnel, a doctor-centered model of care, and a large number of medical university students, we see the prospect of involving students in working with the population. Our study aimed to evaluate the effectiveness of patient education conducted by the volunteer students at the medical university who completed a short specially designed course outside the main educational program. In this article, we attempted to assess whether visits by trained volunteers to patients with CVD reduced depression and anxiety.

## 2. Materials and Methods

### 2.1. Study Design

A randomized controlled trial was conducted with two groups of patients followed up for 12 months, comparing the educational intervention group with the usual outpatient follow-up practice for CVD patients after acute conditions. Patients were recruited from the cardiology department of the university hospital, Medical Center of the Non-Commercial Joint Stock Company West Kazakhstan Marat Ospanov Medical University in the northwest of Kazakhstan. Ethical approval was obtained from the Local Ethical Committee of the West Kazakhstan Marat Ospanov Medical University. The study was conducted as part of the scientific and technical project “Building the Capacity of Medical Education Technologies and Research in Family Medicine in Kazakhstan”, with a grant from the Science Committee of the Ministry of Science and Higher Education of the Republic of Kazakhstan (grant no. AP09260428).

### 2.2. Participants

All patients who met the inclusion criteria and agreed to participate in the study were randomized into intervention and control groups in a 1:1 ratio using a computer random number generator. The baseline characteristics of the study participants are presented in Table 1.

The study participants were assessed using the Russian-validated Hospital Anxiety and Depression Scale (HADS) [17] and the Hamilton Rating Scale for Depression (HDRS) [18] before the start of the study and after 12 months. The HADS measures the symptoms of anxiety (7 points) and depression (7 points). The items are rated on a 4-point (0–3) scale, and higher scores indicate increased levels of stress. The scores for each subscale range from 0 to 21 and can be classified as normal (0–7), mild (8–10), moderate (11–14), and severe (15–21). A HADS score of 8 or higher is considered the threshold for mild clinical symptoms, and provides optimal sensitivity and specificity for case detection [19]. HDRS scores of 0 to 7 are considered normal, 8–16 indicate mild depression, 17–23 indicate moderate depression, and scores greater than 24 indicate severe depression; the maximum score is 52 points on a 17-point scale [20]. A clinically significant reduction in anxiety and depression was defined as a reduction of at least 3 points on the HDRS scale, 1.7 points on the HADS scale, and/or a standardized mean difference (SMD) greater than 0.3 [21,22].

### 2.3. Interventions

Participation in study groups does not preclude the possibility of adding treatment for severe anxiety and depression, which may be necessary and offered by mental health professionals as part of routine outpatient follow-up. This is possible if the general practitioner or cardiologist suspects the presence of a mental disorder and refers the patient to a psychiatrist or at least a psychologist. However, the current clinical protocol in Kazakhstan does not include any recommendations for the identification and management of patients with comorbid depression or anxiety.

The current practice of dispensary observation of patients receiving CR (basic/standard) care in both groups of the study participants includes a mandatory amount of assistance as part of outpatient treatment at the patient’s place of residence, as prescribed by the orders of the Ministry of Health of the Republic of Kazakhstan, the clinical protocol:

1. On approval of the rules for organizing the provision of medical care to people with chronic diseases, the frequency and timing of observation, the mandatory minimum and frequency of diagnostic studies. Order of the Minister of Health of the Republic of Kazakhstan dated 23 October 2020 no. RK HM-149 /2020 (registered in the Register of State Registration of Regulatory Legal Acts under no. 21513). Available at: https://adilet.zan.kz/rus/docs/V2000021513 (accessed on 30 October 2020).
2. On approval of the rules for the provision of medical rehabilitation. The order of the Minister of Healthcare of the Republic of Kazakhstan dated 7 October 2020 no. KR HM- 116/2020 (registered in the Register of State Registration of Regulatory Legal Acts under no. 21381). Available at: https://adilet.zan.kz/rus/docs/V2000021381 (accessed on 13 October 2020).
3. Clinical protocol for medical rehabilitation stage three “outpatient rehabilitation ii” profile “cardiology and cardiac surgery” (adults). Recommended by the Expert Council of the RSE on REM “Republican Center for Health Development” of the Ministry of Health and Social Development of the Republic of Kazakhstan dated 12 December 2014 protocol no. 9. Available at: https://endovascular.kz/ru/rekomendatsii/klinicheskie-protokoly-mz-rk/tretij-etap-ambulatornaya-reabilitatsiya-ii-profil-kardiologiya-i-kardiokhirurgiya-vzroslye (accessed on 13 October 2020).

Polyclinics have the necessary specialists, and schools for patients are held, with examinations and medications (antihypertensive, antiischemic, statins, antiplatelet agents) provided free of charge for insured patients, but no antidepressants, psychotherapists, and often psychiatrists. Psychologists have a pedagogical education and are not trained to deal with sick people. Patients subject to planned hospitalization and inpatient rehabilitation (on average 10 days) can also receive these services free of charge, in accordance with regulatory legal acts. Patients subject to planned hospitalization and inpatient rehabilitation (on average 10 days) can also receive these services free of charge, in accordance with regulatory legal acts.

### 2.4. Educational Intervention

The standard care of patients after myocardial infarction, according to the order, lasts 12 months. Our educational intervention was delivered alongside usual CR for a year, consisting of a minimum of 12 weekly volunteer visits 60 min or longer during the first three months, followed by visits at least once a month. A detailed description of the intervention is provided in our previous article [23]. Volunteers who completed a specially designed training made home visits to patients in need of CR for education, information, support, and motivational interviews. Each visit lasted 60 min: the first part of the visit included gathering information, a joint discussion about the implementation of specialist treatment recommendations, success in lifestyle changes, and an assessment of the patient’s physical condition by the volunteer with an explanation of the results; the second part was training on a specific topic (visit 1—medications, visit 2—smoking cessation or weight loss, depending on the presence of a risk factor in the patient, etc.). During the training, volunteers used their gadgets to demonstrate visual materials. Visits were conducted weekly during the first three months, followed by once-a-month visits, with telephone counseling available more frequently upon the patient’s request. The educational visits covered topics such as lifestyle and risk factor management, exercise, medication, symptoms, and laboratory control. Additionally, volunteers conducted motivational interviews and provided patient support.

Volunteers completed a 5-day training course where they were trained to assess both the physical and mental states of patients. They were also taught about technologies for conducting individual training on nutrition, physiotherapy exercises, self-management, the mechanism of action and taking medications, and conducting a motivational interview. At the end of the course, the volunteers underwent an exam to assess their newly acquired skills, received instructions on how to work with patients, communicate and receive feedback from them, and how to liaise with patients’ district doctors and mentor-curators of the research group.

### 2.5. Collection of Information

Information was collected through two patient assessments: the baseline (pre-CR) assessment and the assessment conducted 12 months after randomization.

### 2.6. Statistical Analysis

The sample size was calculated using WinPepi software with alpha- and beta-errors set at 5% and 20%, respectively, based on the expected clinically important differences. Numeric variables were presented as the mean ± standard deviation (SD), while categorical variables were presented as absolute numbers and percentages. Mann–Whitney tests were used to compare continuous variables between independent groups, while Wilcoxon tests were used for paired observations. Pearson’s chi-squared tests were used for analyzing categorical variables, and McNemar tests were applied for paired comparisons of dichotomous data. In addition to significance testing, effect sizes were calculated for all tests performed. An SMD was used to compare the two mean values, with a value of 0.20 indicating a small effect, 0.50 indicating a medium effect, and 0.80 indicating a strong effect. All differences were considered significant at *p* < 0.05. All calculations were performed using SPSS software (version 25; IBM SPSS Inc., Chicago, IL, USA).

## 3. Results

**Figure 1:**
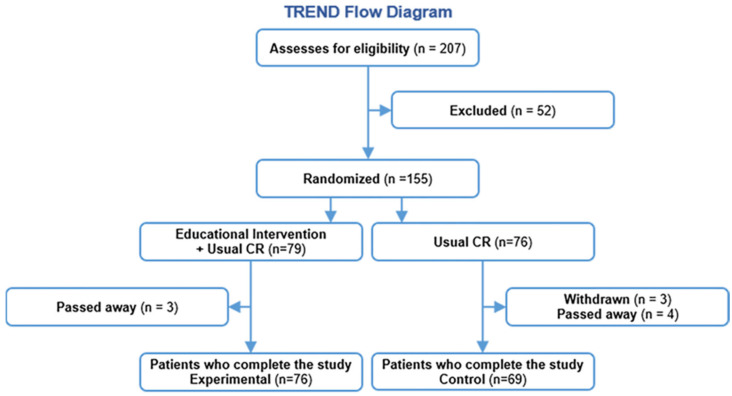
Trend diagram of a randomized controlled trial for educational intervention effects on depression and anxiety in patients after myocardial infarction.

Between 1 April 2019 and 31 March 2020, a total of 207 patients were referred for CR and screened for eligibility (Figure 1). Of these, 22 patients did not meet the inclusion criteria, 30 withdrew, and 79 were randomized to the intervention group and 76 (49%) to the control group.

Table 1 presents the demographic and clinical data for the sample at baseline. The groups were well balanced for all measured variables, including the severity of physical illness and the prevalence of mental disorders. There was no major depressive disorder in either group. Changes after a year are presented in Table 2, Table 3, Table 4 and Table 5. Medications taken by the patients in both groups during the year (antiplatelet agents, angiotensin converting enzyme inhibitors, angiotensin II receptor blockers, beta-blockers, calcium channel blockers, diuretics, aldosterone antagonists, nitrates, statins, antisecretory drugs) did not include antidepressants.

The intervention improved lifestyle measures, including a reduced body mass index, waist circumference, and smoking. It also improved cardiovascular performance, indicated by the systolic blood pressure (SBP), diastolic blood pressure (DBP), heart rate (HR), EF, and the 6 min test. Furthermore, it had a positive impact on indicators of lipid metabolism, such as total cholesterol, low-density lipoprotein (LDL), and high-density lipoprotein (HDL). In addition, the intervention resulted in a decrease in hospital admissions (RR 0.18, 95%CI 0.04–0.79; NNT 8.4), as shown in Table 2 and Table 3. The number of days after discharge to emergency hospitalization associated with deterioration in the experimental group was 45 and 212 days (median 151 days), and in the control group from 15 to 308 days (median 108 days, interquartile interval 83–145 days), *p* = 0.98.

The percentage of patients achieving the target SBP ≤ 120 mmHg and DBP ≤ 80 mmHg was significantly higher in the intervention group (77.6% (95% CI 68–87%) and 97.4% (95% CI 94–101%)), with an RR of 2.14 (95% CI 1.5–2.9%, NNT 2.4), compared to the control group (36.2% (95% CI 25–48%) and 65.2% (95% CI 54–76%)), with an RR of 1.4 (95% CI 1.2–1.7%) and an NNT of 3.4.

The level of total cholesterol decreased significantly in the experimental group compared to the control group (Table 2 and Table 3). However, the number of patients who reached the target LDL level < 1.8 mmol/L was low in both groups, with six patients in the experimental group and five patients in the control group. The percentage of patients who achieved at least a ≥50% reduction in cholesterol levels between 1.8 and 3.5 mmol/L was statistically significantly higher in the experimental group (14.5% (95% CI 7–22%) compared to 1.4% (95% CI 1–4%)), with an RR of 9.9 (95% CI 1.3–75.3%).

In the intervention group, there was a decrease not only in the body mass index (BMI) (Table 2 and Table 3), but also in body weight, with an average reduction of −3.3 kg (−3.9%) compared to the baseline (*p* < 0.001). Conversely, the control group experienced an increase in BMI and body weight by 2.3% (from 78.4 (12.7) kg to 79.8 (12.5) kg). Tolerance to physical activity in the experimental group increased by 24.9%, whereas in the control group, it only increased by 10.3%.

After 12 months, the number of smokers in the experimental group decreased by 32.9% (from 56.6 to 23.7%), while in the control group, it decreased by 5.8% (from 42 to 36.2%), resulting in an RR of 0.65 (95% CI 0.39–1.09). Among those who continued to smoke, there was a reduction in the number of cigarettes smoked (Table 2 and Table 3). However, the effect on death (RR 0.68, 95%CI 0.15–2.9; NNT 54.1), recurrent myocardial infarction (RR 0.34, 95%CI 0.09–1.2; NNT 13.1), and stroke (RR 0.23, 95%CI 0.03–1.98; NNT 22.3) tended to decrease, although the results were not statistically significant.

The HADS depression scores in both groups before the intervention were statistically similar (3.04 ± 3.7 in the intervention group versus 3.83 ± 3.89) in the control group, *p* = 0.07), as were the Hamilton Rating Scale for Depression (HRDS) scores (4.2 ± 4.7 vs. 4.5 ± 5.3, *p* = 0.50). HADS anxiety scores also did not differ significantly (3.9 ± 3.2 versus 4.5 ± 3.4, *p* = 0.35, in the intervention and control groups, respectively).

After 12 months, the mean HADS depression scores were 1.1 ± 1.7 in the intervention group versus 2.6 ± 2.9 in the control group, *p* < 0.0001, the HRDS scores were 1.5 ± 1.7 versus 3.1 ± 4.3, *p* = 0.009, and the HADS anxiety scores were 1.9 ± 1.5 versus 3.0 ± 1.8, *p* = 0.0002, in the intervention and control groups, respectively.

In the intervention group, the decrease in anxiety and depression on both scales exceeded the decrease in the control group, although the comparison of the differences was not statistically significant (Table 4). The group difference on the Hamilton scale at 12 months was −1.29 in favor of the intervention group (95% CI, −0.7 to −1.88); SMD 0.36 (95% CI, 0.03 to 0.69). The group difference on the HADS anxiety scale after 12 months was −0.5 in favor of the intervention group (95% CI, from −0.09 to −0.9); SMD 0.2 (95% CI, −0.13 to 0.5). The group difference on the HADS depression scale at 12 months was −0.7 in favor of the intervention group (95% CI, −0.22 to −1.17); SMD 0.24 (95% CI, −0.09 to 0.56).

The proportion of patients with anxiety on the HADS scale in the intervention group decreased from 18.4% to 1.3% (*p* = 0.0002), while in the control group, it decreased from 18.8% to 5.8% (*p* = 0.001). The proportion of patients with depression in the intervention group decreased from 15.8% to 0 (*p* = 0.0007), and in the control group, it decreased from 14.5% to 2.6% (*p* = 0.002), according to the HADS and HRDS scales, respectively. The development of depression during the year was observed in two patients in the control group who did not initially have depression, whereas positive dynamics were observed in 100% of cases in the intervention group. However, no significant differences were found between the changes (Table 5). The safety and adverse events associated with the intervention were monitored throughout the trial and were not observed.

## 4. Discussion

In this article, we report the results of an educational intervention in patients enrolled in an outpatient CR program. By the end of the study, an SMD ≥ 0.8 was observed for the BMI, wrist circumference, the number of cigarettes smoked, blood lipids other than triglycerides, and the 6 min walk test. The weight loss reported in cardiac rehabilitation programs in the scientific literature aligns with the findings of our study [24]. Regarding smoking, our study demonstrated lower results (33% reduction) compared to those reported by other authors (53–58%) [25,26]. Based on our results, the intervention has the potential to significantly reduce LDL and TC levels, while increasing serum HDL levels. Similar findings are supported by the Wu et al. [27] systematic review.

The minimum clinically significant difference for the six-minute walk test in coronary artery disease (CAD) patients after acute coronary syndrome is considered to be 25 m [28]. However, recent studies have shown a substantially greater improvement in exercise tolerance, such as the study by Gao et al. [29], which reported an increase of 200 m. In our study, we also observed a notable improvement in this indicator, with an average increase of 88 m, representing a 25% improvement over the baseline value.

One year later, in the main group, anxiety and depression on the HADS scale decreased by 2.0 and 1.9 points, respectively (*p* < 0.05). Depression on the HDRS scale decreased by an average of 1.29 points (*p* < 0.05), while in the control group, there was no clinically significant decrease, and 5.8% of patients remained moderately to severely depressed. The effect size for HDRS depression at 12 months was 0.36. The results show a slight improvement in performance in the CR control group, which contrasts with a more significant improvement in the main group. Different authors consider a change in HDRS-17 by 3–6 points to be a clinically significant decrease in the level of depression [30,31]. For anxiety and depression, a decrease of 1.7 points is considered to be the minimum clinically significant difference for HADS [22].

The UK National Institute for Health and Care Excellence (NICE) considers a reduction of 3 points on the HDRS of 17 points as a criterion for the effectiveness of depression treatment [32]. Some authors recognize an SMD of about 0.3 as another criterion of minimal clinical significance in comparative studies [21]. However, these criteria can be called conditional because obtaining such values is not always a threshold. For example, people with less severe depression require smaller absolute reductions in HDRS scores for a clinically meaningful difference. In addition, some of the symptoms assessed by the HDRS may be a manifestation of other patient conditions other than depression, such as medical illness, drug side effects, and may persist despite improved mental status [21]. This is supported by the observed discrepancy between the HDRS score and remission, as assessed by patients [33].

Clinical relevance remains uncertain even for pharmacological antidepressants. Numerous meta-analyses show that the SMD of antidepressants in the treatment of depression is 0.3. The average reduction in HDRS scores associated with antidepressant use is 2 points [21,31]. Psychotherapy has the same clinical value as antidepressants, i.e., 0.3–0.4 on the difference in effects [34]. Overall, the Cochrane Review found that both psychological and pharmacological interventions have low certainty evidence for an effect on depression in patients with CAD due to the small number of outcome trials and the heterogeneity of the study populations and interventions [35].

CR has been shown to reduce mortality and readmissions and improve the QoL [36]. However, its effect on anxiety and depression has been demonstrated only in combination with CR and psychotherapy. The PATHWAY study, which added group metacognitive psychotherapy sessions to a standard CR program, showed significant improvement in both depressive and anxiety symptoms [37].

### 4.1. Goals of CR Programs

The key goal of CR programs is to improve physical health and the QoL, and to equip and support people to develop the necessary skills for successful self-management [38,39,40]. Psychological components are not standardized and vary across healthcare resources. They include counseling, relaxation, meditation, stress management, cognitive psychotherapy, social support, help, and communication channels.

### 4.2. Psychological Component in Outpatient CR

In our program of outpatient CR, the psychological component included regular informative assessments of physical conditions (such as BP, HR, exercise tolerance, and interpretation of laboratory and instrumental examinations), training in self-management methods aimed at improving CVD risk profiles, increasing physical activity, and providing support and continuous feedback from the patient. There was no psychotherapy, psychological counseling, stress management, or consultations with a psychiatrist or a psychotherapist. Improvement in physical condition, increased physical activity, and clear patient-oriented recommendations, in our opinion, ensured the results obtained.

It is known that multicomponent self-management support strategies [41] and patient-centered approaches [42], including support, involvement, active listening to patients, and coordination of care, improve clinical treatment outcomes for chronic patients, including mental state and the quality of life [43]. They increase patients’ confidence in the healthcare system and in the medical workers themselves, self-confidence, reduce anxiety associated with helplessness, and also form positive beliefs in their needs and the value of their lives. Leveraging the patient’s potential for self-care and involvement in decision-making increases commitment to adherence to the recommendation and lifestyle changes, thus ensuring the quality of care.

### 4.3. Strengths and Limitations

Strengths of the study include a structured intervention, randomization, and the use of long-term follow-up. The sample of this study is representative of patients with CVD. For example, Choo et al. [7] reported that 74% of CR patients were male, and the mean and SD of the age was 57 ± 8.8, which is comparable to our study by sex and age. Wells et al. [37] reported that half of the patients had concomitant arterial hypertension, and 23% had diabetes mellitus, which is similar to our study in which 58% of patients had hypertension, and 23% had type 2 diabetes.

Our study has some limitations that need to be addressed in the future. Our sample had a small size, which likely limited our ability to detect little effects of the intervention. Nevertheless, the results seem to reflect the true state of affairs, as other studies show. The Wells et al. [2] study had 332 participants in two groups, the Choo et al. [7] study had 194 patients, Ayasrah et al. [44] had 186 patients, while Sharif et al. [45] had 80 participants. There were 145 patients in our study. The findings were drawn predominantly from a Kazakh population, and replication is needed in other ethnic groups. Patients also received moderate doses of atorvastatin (40 mg, 30 tablets per month, limited to government procurement for free dispensation to patients), which resulted in suboptimal LDL levels achieved. Recruitment was carried out at a single site. This may limit the generalizability of the results. We did not use an intent-to-treat analysis recommended in RCTs, although there was a low dropout rate (6.5%) in our study, considering that up to 20% of dropout during a trial can be deemed acceptable [46]. This study is an open-label trial, in which treatment bias cannot be completely excluded. Thus, future large-scale studies are needed to replicate and validate our findings.

### 4.4. Practical Significance

The practical significance of our study is likely to be different for various healthcare systems and medical education. In our country, students of medical HEIs and medical colleges can be potential system assistants who can interact closely with patients. However, there is not enough staff in the primary care system to better cover the population with effective medical services, not only for the rehabilitation of patients with CVD, but also for patients with other chronic diseases, palliative care, and preventive activities. Our study is one of its kind that has evaluated the effectiveness of using students for healthcare practice.

## 5. Conclusions

Educational intervention in routine CR appears to be safe and effective in reducing anxiety and depression compared to conventional care. Benefits appeared to be stable over the 12-month follow-up period, and the effect size is comparable to the best existing results from research on depression. Educational interventions for cardiac rehabilitation that do not increase the incidence of adverse events are effective and safe, offering notable clinical benefits in terms of improving exercise endurance, reducing hospitalization, and managing risk factors. Lowering blood pressure and LDL, and smoking cessation have been proven to improve the prognosis for cardiovascular events. It is very important to best deliver this knowledge to patients, to involve them in the decision-making process in order to achieve the goals of cardiac rehabilitation. In relation to depression and anxiety, the method we tested was found to be a non-invasive, non-pharmacological, affordable way without complications, and complementary to already-established patient recovery technologies. We believe that the intervention could be included in routine CR to significantly improve psychological outcomes in patients with CVD and offer added value over standard CR.

## Figures and Tables

**Table 1:** Demographic and clinical indicators of patients in a randomized controlled trial for educational intervention effects on depression and anxiety after myocardial infarction.

Index	Experimental Group (n = 76)	Control Group (n = 69)	*p*-Value
Age, years (range)	58 (52–68)	59 (54.5–69)	0.24
Systolic blood pressure, mmHg (range)	140 (130–150)	130 (120–150)	0.37
Diastolic blood pressure, mmHg (range)	85 (80–90)	80 (80–90)	0.40
Heart rate, bpm (range)	75 (68–85)	78 (70–80)	0.44
Cholesterol, mmol/L (range)	5.6 (4.6–6.5)	5.1 (4.2–6.5)	0.15
Triglycerides, mmol/L (range)	1.2 (0.9–1.8)	1.2 (0.9–1.8)	0.82
High-density lipoprotein, HDL, mmol/L (range)	1.1 (0.9–1.3)	1.2 (1.1–1.2)	0.79
Low-density lipoprotein, LDL, mmol/L (range)	3.5 (2.9–4.1)	3.2 (2.5–4.2)	0.44
Glucose, mmol/L (range)	6.7 (5.5–8.9)	6.5 (5.2–7.9)	0.15
Creatinine, µmol/L (range)	73 (66–89)	79 (67.6–92.9)	0.37
Smoked, n (%)	43 (56.6%)	29 (42.0%)	0.08
Hypertension Grade 3, n (%)	44 (57.9%)	44 (63.8%)	0.82
Recurrent myocardial infarction, n (%)	12 (15.8%)	17 (24.6%)	0.18
Stroke/Transient ischemic attack, n (%)	7 (9.2%)	8 (11.6%)	0.57
Diabetes mellitus, n (%)	18 (23.7%)	13 (18.8%)	0.47
Ejection fraction by echocardiography, n (%)	50 (46–54)	50 (45–53)	0.50
6 min walk test, n (%)	365 (335–400)	370 (330–440)	0.62
Gender			0.12
Male, n (%)	58 (73.4%)	61 (83.6%)	
Female, n (%)	21 (26.6%)	12 (16.4%)	
Place of residence			0.44
City, n (%)	47 (59.5%)	42 (57.5%)	
Rural area, n (%)	19 (24.1%)	23 (31.5%)	
Suburb, n (%)	13 (16.5%)	8 (11.0%)	
Marital status			0.50
Married, n (%)	61 (77.2%)	60 (82.2%)	
Divorced/Widower, n (%)	16 (20.3%)	10 (13.7%)	
Single, n (%)	2 (2.5%)	3 (4.1%)	
Education			0.57
Secondary, n (%)	36 (45.6%)	37 (51.4%)	
Vocational, n (%)	22 (27.9%)	21 (29.2%)	
Higher, n (%)	21 (26.6%)	14 (19.4%)	
Employment			0.70
Unemployed, n (%)	34 (43.0%)	34 (46.6%)	
Manual labor, n (%)	31 (39.2%)	24 (32.9%)	
Mental labor, n (%)	14 (17.7%)	15 (20.6%)	
Hospital Anxiety and Depression Scale (HADS), anxiety, n (%)	14 (18.4%)	13 (18.8%)	0.98
Subclinical anxiety, n (%)	9 (11.8%)	8 (11.6%)	
Clinical anxiety, n (%)	5 (6.6%)	5 (7.3%)	
Hospital Anxiety and Depression Scale (HADS), depression, n (%)	12 (15.8%)	10 (14.5%)	0.97
Subclinical depression, n (%)	6 (7.9%)	5 (7.2%)	
Clinically significant depression, n (%)	6 (7.9%)	5 (7.2%)	
Hamilton, depression, n (%)	11 (14.5%)	10 (14.5%)	0.66
Mild depressive disorder, n (%)	5 (6.6%)	5 (7.3%)	
Moderate depressive disorder, n (%)	3 (4.0%)	1 (1.5%)	
Severe depressive disorder, n (%)	3 (4.0%)	3 (4.4%)	

**Table 2:** Mean ± standard deviation (SD) of characteristics of the patients after 12 months in the experimental and control groups of the randomized controlled trial for educational intervention effects on depression and anxiety after myocardial infarction.

Indices	Experimental (n = 76)	Control (n = 69)	*p*-Value
Body mass index, BMI, kg/m^2^	27.1 ± 4.8	28.3 ± 3.8	0.00
Wrist circumference, cm	97.2 ± 13.1	102.2 ± 12.2	0.01
Cigarettes smoked per day, n	1.9 ± 4.2	6.2 ± 8.9	0.00
Systolic blood pressure, mmHg	119.0 ± 8.3	132.2 ± 15.7	0.00
Diastolic blood pressure, mmHg	75.9 ± 5.9	82.2 ± 8.6	0.00
Heart rate, bpm	63.1 ± 3.4	68.6 ± 7.4	0.00
Cholesterol, mmol/L	4.1 ± 0.8	4.9 ± 1.2	0.00
Triglycerides, mmol/L	1.4 ± 0.5	1.5 ± 0.7	0.10
High-density lipoprotein, HDL, mmol/L	1.2 ± 0.2	1.0 ± 0.2	0.00
Low-density lipoprotein, LDL, mmol/L	2.5 ± 0.8	3.1 ± 0.9	0.00
Ejection fraction by echocardiography, %	53.6 ± 6.5	49.7 ± 6.9	0.00
6 min walk test, m	442.6 ± 71.8	372.2 ± 101.4	0.00
Death, n (%)	3.0 ± 3.8%	4.0 ± 5.4%	0.62
Recurrent myocardial infarction, n (%)	3.0 ± 3.8%	8.0 ± 10.9%	0.08
Stroke/Transient ischemic attack, n (%)	1.0 ± 1.2%	4.0 ± 5.4%	0.14
Hospitalization, n (%)	2.0 ± 2.5%	10.0 ± 13.7%	0.01

**Table 3:** Mean ± standard deviation (SD) of the differences between the values after 12 months and at the initiation of the study in the experimental and control groups of the randomized controlled trial for educational intervention effects on depression and anxiety after myocardial infarction.

Indices	Intervention Group	Control Group	*p*-Value	Effect Size (95% CI)
Body mass index, kg/m^2^	−1.1 ± 1.0	0.4 ± 1.1	<0.001	−1.49 (96% CI −1.8; −1.12)
Waist circumference, cm	−3.4 ± 2.4	1.8 ± 2.7	<0.001	−2.08 (96% CI −2.5; −1.7)
Number of cigarettes a day, n	−10.6 ± 11.2	−2.6 ± 7.2	<0.001	−0.8 (−1.18; −0.5)
Systolic blood pressure, mmHg	−18.7 ± 18.4	−4.9 ± 25.5 * p = 0.11	<0.001	−0.63 (96% CI −0.96; −0.3)
Diastolic blood pressure, mmHg	−8.16 ± 9.4	−1.3 ± 12.4 * p = 0.39	<0.001	−0.63 (96% CI −0.96; −0.3)
Heart rate, bpm	−12.9 ± 11.9	−8.2 ± 11.2	0.01	−0.4 (95% CI −0.7; −0.07)
Cholesterol, mmol/L	−1.57 ± 1.2	−0.39 ± 1.2	<0.001	−0.98 (95% CI −1.3; −0.6)
Triglycerides, mmol/L	−0.08 ± 0.7 * p = 0.38	0.13 ± 0.7 * p = 0.16	0.10	−0.3 (95% CI −0.6; 0.03)
High-density lipoprotein, HDL, mmol/L	0.08 ± 0.3	−0.05 ± 0.2 * p = 0.15	0.006	0.46 (95% CI 0.13; 0.8)
Low-density lipoprotein, LDL, mmol/L	−0.9 ± 1.1	−0.2 ± 0.9 * p = 0.07	<0.001	−0.7 (95% CI −1.02; −0.36)
Ejection fraction by echocardiography, %	4.2 ± 4.9	0.7 ± 4.6 * p = 0.19	<0.001	0.72 (95% CI 0.39; 1.06)
6 min walk test, m	88.1 ± 28.9	34.8 ± 28.0	<0.001	1.9 (95% CI 1.5; 2.3)

* Changes were not statistically significant.

**Table 4:** Mean ± standard deviation (SD) of categorical characteristics in the experimental and control groups at the beginning and end of the randomized controlled trial for educational intervention effects on depression and anxiety after myocardial infarction, as well as their changes.

Characteristics *	Experimental Group, n = 76			Control Group, n = 69			Experimental Group Changes	Control Group Changes	*p*-Value ^2^
	Before	After	*p*-Value ^1^	Before	After	*p*-Value ^1^			
HADS anxiety	3.9 ± 3.2	1.9 ± 1.5	0.0002	4.5 ± 3.4	3.0 ± 1.8	<0.001	−2.0	−1.5	0.24
HADS depression	3.0 ± 3.7	1.1 ± 1.7	<0.0001	3.8 ± 3.9	2.6 ± 2.9	<0.001	−1.9	−1.2	0.21
HDRS depression	4.2 ± 4.7	1.5 ± 1.7	0.008	4.5 ± 5.3	3.1 ± 4.3	<0.001	−2.7	−1.4	0.23

*Abbreviations: HADS—Hospital Anxiety and Depression Scale; HDRS—Hamilton Rating Scale for Depression; ^1^ According to the Wilcoxon test for paired populations, ^2^ according to the Mann–Whitney test for unpaired populations.

**Table 5:** Frequency of categorical features (outcome—yes/no) in the experimental and control groups at the beginning and end of the randomized controlled trial for educational intervention effects on depression and anxiety after myocardial infarction, as well as their changes.

Characteristics	Experimental Group			Control Group			*p*-Value ^2^
	Before, Positive Outcome	Before, Negative Outcome	*p*-value ^1^	Before, Positive Outcome	Before, Negative Outcome	*p*-Value ^1^	
HADS anxiety			<0.001			0.008	0.19
After, Negative outcome	13	62		9	56		
After, Positive outcome	1	0		4	0		
HADS depression			0.002			0.04	0.07
After, Negative outcome	12	64		8	58		
After, Positive outcome	0	0		2	1		
HDRS depression			0.008			0.68	0.06
After, Negative outcome	9	65		4	57		
After, Positive outcome	2	0		6	2		

^1^ Comparison using the McNemar test for paired populations, ^2^ comparison using Pearson’s chi-square test for unpaired populations.

## Data Availability

All data generated or analyzed during this study are included in this published article.
